# Development of a codebook for the narrative analysis of in‐hospital trauma interviews of patients following stroke

**DOI:** 10.1002/jts.23106

**Published:** 2024-11-01

**Authors:** Corinne Meinhausen, Anusha Fatehpuria, Jaifreen Bhangu, Donald Edmondson, Ian M. Kronish, Patrick Wilson, Jennifer A. Sumner

**Affiliations:** ^1^ Department of Psychology University of California Los Angeles California USA; ^2^ Center for Behavioral Cardiovascular Health Columbia University Irving Medical Center New York New York USA

## Abstract

Given their sudden onset and life‐threatening consequences, strokes and transient ischemic attacks (TIAs) can trigger posttraumatic stress disorder (PTSD). To gain a deeper understanding of the potential influence of factors in patients’ descriptions of these medical events on PTSD, we conducted a standardized trauma interview with a convenience sample of patients hospitalized for suspected stroke/TIA (*N* = 98) to assess the details and emotional experience of the stroke/TIA event. Three researchers reviewed the interviews and the research literature on risk and protective factors for PTSD. From this analysis, a codebook with descriptions, examples, and scoring protocols for eight Likert scale, two categorical, and four binary codes was developed. Upon demonstrating sufficient interrater reliability, the research team scored all narratives. Three superordinate themes were identified in the analysis: distress (e.g., fear, helplessness), potential protective factors (e.g., positive expectancies, concern for loved ones), and level of detail (e.g., somatic detail, emotional detail). Differences in perceptions, themes, and expectations emerged in the narratives, indicating a wide range of responses following stroke/TIA. Additionally, patient age was negatively correlated with scores for the fear, *r* = −.34, *p* < .001, and negative consequences, *r* = −.24, *p* = .018, codes and positively associated with the likelihood of having positive expectancies, *OR* = 1.05, 95% CI [1.00, 1.10], *p* = .039. These findings provide a more comprehensive understanding of how patients reflect on their experiences post–stroke/TIA and can inform future research on the contributions of trauma narrative characteristics and emotional responses to PTSD risk.

Strokes are a leading cause of global disability, with more than 100,000,000 people worldwide living as stroke survivors (Feigin et al., 2022). Strokes are often associated with reduced functional ability, which can result in limited activity and independence, as well as a loss of the sense of self (Crowe et al., 2016). As a result, psychological health and health‐related quality of life may be negatively impacted in the aftermath of these events. Feelings of anxiety, panic, guilt, and sadness are common following a stroke, and emotional distress is often cited as one of the most impactful challenges both patients and their caregivers face during recovery (McCurley et al., 2019). Higher levels of emotional distress following a stroke are, in turn, related to a heightened risk of stroke recurrence and mortality (Carod‐Artal & Egido, 2009).

Furthermore, the often sudden, uncontrollable, and potentially life‐threatening nature of stroke events may induce symptoms of posttraumatic stress disorder (PTSD). Meta‐analytic evidence suggests that 23% of individuals who experience a stroke or transient ischemic attack (TIA) develop PTSD symptoms in the year following the event, with 11% continuing to experience PTSD symptoms after the first year (Edmondson et al., 2013). Moreover, PTSD poststroke has been linked to poorer outcomes, including lower medication adherence and worse quality of life (Kronish et al., 2012; Stein et al., 2018). Although cross‐sectional studies have identified some demographic and medical risk factors for the development of PTSD following stroke/TIA, including younger age, higher level of stroke severity, and female gender, findings have been inconsistent across studies (Garton et al., 2017). A smaller, yet growing, number of studies have explored the psychological and cognitive factors that may increase the risk for PTSD symptom development poststroke. For example, one recent study found that individuals who developed PTSD symptoms in response to a prior traumatic event had more severe stroke‐induced PTSD symptoms at 9–13 months poststroke compared to those without prior trauma exposure and those who reported prior trauma exposure but did not develop PTSD symptoms, after adjusting for age and sex (Kronenberg et al., 2021). Individuals with a higher degree of negative appraisals of themselves and the world have also been shown to be at heightened risk for PTSD symptom development poststroke (Bruggimann et al., 2006; Field et al., 2008), mirroring results from studies in individuals with PTSD following nonmedical trauma exposure (Ehlers & Clark, 2000).

Some research exploring the associations between an individual's posttrauma emotional and cognitive response and the development and severity of PTSD symptoms has involved an analysis of trauma narratives (i.e., an individual's description of their traumatic experience through written or spoken prompts or semistructured interviews). Studies have shown that a higher degree of somatic and sensory details (e.g., Beaudreau, 2007; Jones et al., 2007) and negative and visceral emotional detail (e.g., Eid et al., 2005; Greenhoot et al., 2013) when describing the traumatic experience are associated with higher levels of PTSD symptom severity in individuals following nonmedical trauma. Notably, many of the trauma narratives included in these studies were provided years or decades following trauma exposure. Although little research has examined themes in trauma narratives obtained during the acute aftermath of trauma, one study that used computerized text analysis of narratives 18 days posttrauma found that the use of fewer cognitive elaboration words and more life‐threat and self‐focused words predicted higher PTSD symptom levels 6 months later (Kleim et al., 2018). Furthermore, more research that specifically considers PTSD and relevant processes when examining psychological responses in the acute period following a stroke/TIA is needed (Juth et al., 2018).

In the current study, we performed an analysis of trauma narratives of stroke/TIA survivors obtained in‐hospital in the days following a stroke/TIA. In our analysis of the narratives, we employed an abductive thematic approach (Thompson, 2022), which incorporates the influence of both the empirical narrative data and extant theoretical understanding regarding risk and protective factors for PTSD and factors associated with stroke/TIA recovery. In addition to identifying key themes in the trauma narratives, we aimed to characterize the range of responses across patients and compare the relative occurrence of the themes by demographic factors.

## METHOD

### Participants

Participants were enrolled in a longitudinal cohort study of risk factors for and consequences of poststroke PTSD (Reactions to Acute Care and Hospitalization for Suspected Stroke [REACH Stroke] study). The REACH Stroke study was introduced to potentially eligible patients during hospitalization for suspected stroke/TIA at the NewYork‐Presbyterian/Columbia University Irving Medical Center, an academic medical center located in Manhattan, NY. The inclusion criteria were: English or Spanish fluency, being 18 years of age or older, and having experienced a suspected stroke or TIA per the treating neurologist. Individuals were excluded if they had severe stroke symptoms (National Institutes of Health [NIH] Stroke Scale score greater than 14; Goldstein et al., 1989) and significant aphasia, dysarthria, or cognitive impairment; had severe mental illness or terminal noncardiac medical comorbidities; and/or were not available for follow‐up visits. Eligible patients were provided with study information and gave written informed consent prior to participation. All study components were approved by The Columbia University Irving Medical Center Institutional Review Board (Approval No. AAAQ4612).

Participant characteristics are presented in Table 1. Participants ranged in age from 19 to 87 years (*M* = 60.38 years, *SD* = 16.49), and the sample was diverse with respect to race/ethnicity and educational attainment. Most participants were diagnosed as having a stroke (*n* = 84), with four individuals diagnosed as having a TIA and 10 categorized as having another neurological condition. Approximately 14% of participants (*n =* 14) had a history of stroke or TIA prior to the index event.

**TABLE 1:** Participant sociodemographic and medical characteristics.

Characteristic	*n*	%	*M*	*SD*	Range	Valid *N*
Sociodemographic factors						
Age (years)			60.38	16.49	19–87	98
Gender						98
Male	44	44.9				
Female	54	55.1				
Race/ethnicity						98
Hispanic	39	39.8				
Non‐Hispanic White	20	20.4				
Non‐Hispanic Black	33	33.7				
Non‐Hispanic other	6	6.1				
Educational attainment						98
Less than HS/some HS	22	22.4				
HS graduate	21	21.4				
Trade school/some college	17	17.3				
College graduate	22	22.4				
Graduate school	16	16.3				
Medical factors						
Index event category						98
Stroke	84	85.7				
TIA	4	4.1				
Other	10	10.2				
NIH Stroke Scale score			3.20	3.02	0‐20	88
Prior stroke/TIA history	14	14.3				88
Charlson Comorbidity Index			1.16	1.43	0‐7	98

*Note*: *N* = 98. HS = high school; NIH = National Institutes of Health; TIA = transient ischemic attack.

### Procedure

Between August 2017 and June 2019, participants from the larger cohort study were invited to take part in a psychophysiology substudy during hospitalization for a stroke/TIA event, resulting in a convenience sample of 98 individuals. This subset completed the PhenX Toolkit Baseline and Trauma Challenge Psychophysiological Recordings protocol, consisting of a 2‐min resting baseline followed by a 15‐min standardized trauma interview (PhenX Toolkit, 2024). Trained research coordinators performed the protocol in English or Spanish; although not pertinent to the current study, skin conductance levels were measured continuously during the standardized trauma interview (for a full description of the protocol, see Meinhausen et al., 2023). Research coordinators documented participant responses during the standardized trauma interview, which consisted of 15 open‐ended questions adapted from the Standardized Trauma Interview (Foa & Rothbaum, 1998) used in the PhenX Toolkit protocol to probe for information about the stroke/TIA experience.

Trauma interviews were performed at each participant's bedside, and responses were documented with detailed written notes taken by research coordinators during the interviews. The full interview consisted of a series of structured questions aimed at understanding various aspects of the stroke/TIA event. The interview began by asking participants to describe what happened when they first realized something was wrong during the stroke/TIA event. Participants were then asked about specific episodic details about the stroke/TIA event, including the time of day, their location, how long it lasted (from the moment they first felt they were in danger until they felt safe again), how long ago the event occurred, whether anyone else was with them, and whether they sustained any injuries. The interviewer then queried the participant's concerns about long‐term implications, including whether they anticipated long‐term physical problems or thought they might be permanently disabled or die during the event. Next, participants were asked to describe how helpless, horrified/shocked, afraid/terrified, and in control they felt during the stroke. Finally, interviewers asked whether the participant had experienced similar events before and, if so, how many times. Interview responses provided a brief narrative of participants’ stroke/TIA experiences as well as a description of the degree to which they experienced numerous emotions with relevance to PTSD (e.g., fear, shock, helplessness) in the peritraumatic period.

A team of three researchers (one master's‐level health psychology doctoral student and two undergraduates) began by performing a review of the extant literature on trauma memory narratives and PTSD to identify candidate themes, which were then discussed by the team. Using abductive thematic analysis (Thompson, 2022), the team engaged in repeated reading, coding, and discussion of the written records of the trauma interview to recognize patterns, develop meaningful interpretations, and ultimately construct themes based on both the trauma interview response data and existing theory. Through this process, each member of the research team generated candidate individual codes for the codebook. The team subsequently reviewed each code heuristically, and codes that were deemed insignificant or redundant were removed or consolidated. A codebook comprising 14 codes identified during the abductive thematic analysis was developed, including eight codes scored on a 1–5‐point Likert scale, two categorical codes, and four binary (i.e., “present” or “not present”) codes. Assessed together, these codes suggested three key themes in the empirical data: *distress*, *potential protective factors*, and *level of detail*.

### Measures

#### Codebook development

The research team identified and agreed upon scoring protocols for each code and exemplary responses for each possible rating, which were added to the codebook (see Supplementary Materials for the full codebook). Initial interrater reliability for these codes was first examined in a subset of participants (*n* = 7). Ratings across individuals were compared and discrepancies were discussed, followed by the further refinement of some codes. The revised codebook was then used to assess interrater reliability for five participants, and a two‐way mixed‐effects model (fixed effects of raters) was used to obtain intraclass correlation coefficients (ICCs) measuring the absolute agreement of the three raters for the Likert scale codes. Values ranged from 0.67 to 1.00, indicating moderate‐to‐excellent interrater reliability. Between‐rater disagreement for the binary and categorical scores occurred for one of the 30 codes (3.3%) assessed in this subset. Upon this demonstration of sufficient interrater reliability, records of interview responses for all participants were each scored by two raters; scores were averaged across raters to obtain a final score for the eight Likert scale codes. For the six categorical and binary codes, disagreement between raters occurred in 48 of the 588 codes reviewed (8.2%) and was resolved by a third rater.

#### Participant demographic and clinical characteristics

Participants self‐reported sociodemographic characteristics, including age, gender (male, female), race/ethnicity (Hispanic, non‐Hispanic Black, non‐Hispanic White, other), and educational attainment (less than high school or some high school, high school degree, trade school/some college, college graduate, graduate school). Study neurologists categorized the index medical event resulting in hospitalization as stroke, TIA, or other; the “other” category reflected individuals who were later diagnosed with another neurological disorder (i.e., stroke mimics [Magauran & Nitka, 2012]). Scores from the 11‐item NIH Stroke Scale (Goldstein et al., 1989) were extracted from patients’ medical charts and summed to create a total stroke severity score, which ranged from 0 to 42. Medical condition history, including prior history of stroke/TIA, was extracted from participants’ medical charts by a medically trained research coordinator and used to calculate the Charlson Comorbidity Index (Charlson et al., 1987), a measure of medical comorbidity.

### Data analysis

An exploratory analysis was performed to test differences in the frequency and level of code expression between demographic groups. This exploratory analysis was deemed appropriate given the structured nature of the interviews and the consistency across groups in the environment and circumstances in which the interviews were performed (Guest et al., 2012). Age and gender were selected for analysis, as previous research has identified these variables as potential risk factors for the development of PTSD following stroke (Goldfinger et al., 2014; Kronenberg et al., 2021). There were no missing data for these variables. To test for associations with age, unadjusted correlations were used to compare the codes scored with Likert scales, and logistic regression was used to test associations between age and binary codes. To test for associations with gender, independent‐samples *t* tests were used to compare mean scores on Likert scales between groups. Chi‐squared tests were used to test for associations between gender and both binary and categorical codes; Fisher's exact tests were used when less than 80% of expected cell counts were less than 5. Analyses were performed with SPSS (Version 27.0), and the threshold for statistical significance was set at *p* < .05.

## RESULTS

### Abductive thematic analysis

#### Distress

**FIGURE 1:**
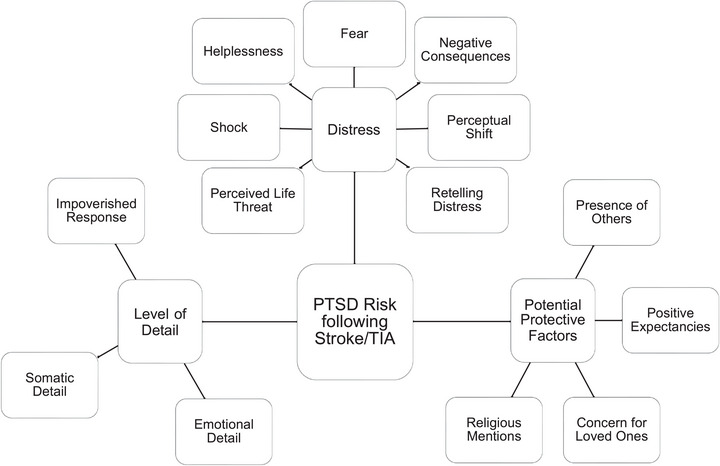
Thematic network analysis of individual thematic codes informing superordinate themes *Note*: PTSD = posttraumatic stress disorder; TIA = transient ischemic attack.

The theme of distress encompassed codes related to perceived life threat, shock, helplessness, fear, negative consequences of the stroke, perceptual shift, and retelling distress (Figure 1). When participants were asked how terrified or afraid they felt during their stroke, replies ranged from “I was very terrified, I couldn't stop thinking ‘stroke, stroke, stroke’” to “No, not afraid. I was with the right people, and I got good medical care.” Distress related to the perceived life threat and negative consequences of the stroke/TIA was also common in the responses, with individuals stating that they feared for their lives during the stroke/TIA event, including statements that they “thought this might be the big one” and “[I] thought I'd die, thought I was gone.” However, other individuals reported that they did not perceive their stroke/TIA as life‐threatening, with one individual stating, “I didn't think I could die, but I was concerned about how to deal with it. I thought that if I could figure out how to deal with it now, I wouldn't be disabled permanently.” In response to the sudden onset of symptoms commonly observed with stroke/TIA, individuals displayed a wide variation in the degree to which they felt shocked. Responses ranged from “I didn't feel shocked or anything, it was what I was used to” to “startled, not shocked” to “really shocked.” Helplessness was among the most commonly expressed feelings, with more than three quarters (*n* = 80) of individuals being coded as feeling at least a moderate degree (i.e., a Likert scale score of 3 or higher) of helplessness during their stroke/TIA event. Individuals described feeling “very helpless, useless and weak” and felt that “there was nothing I could do.”

Definitions and descriptive statistics for the five Likert scale items assessing the degree to which participants reported experiencing different types of distress are shown in Table 2 . Of these 5‐point scales, helplessness had the highest average rating (*M* = 3.54), followed by fear (*M* = 2.89), shock (*M* = 2.79), negative consequences of the stroke (*M* = 2.30), and perceived life threat (*M* = 2.02; for these codes, higher values indicate a higher degree of expression of that indicator of distress). Histograms for these codes revealed that the negative consequences and perceived life threat codes both had a positively skewed distribution, with most individuals (*n* = 51) rated a 1 for their level of perceived life threat and a 2 or below for negative consequences (*n* = 56; Supplementary Figure S1). There were relatively few scores on the low end of the scale for the helplessness code; instead, there were two peaks, with 26 individuals receiving a rating of 3 and 26 receiving a rating of 5. Both the fear and shock codes had bimodal distributions, with the two highest frequencies at each end of the range for these variables, creating a u‐shaped distribution suggesting that many individuals either strongly endorsed or denied these two indicators of distress (Supplementary Figure S1).

**TABLE 2:** Descriptive statistics and definitions of individual thematic codes

Code	Scale type	Definition	*M*	*SD*	*n*	%
Distress						
Fear	Likert	Degree participant reports feeling terrified or afraid during the stroke event	2.89	1.55		
Perceived life threat	Likert	Degree to which the stroke event was perceived as potentially life‐threatening	2.02	1.33		
Negative consequences	Likert	Degree of anticipation of long‐term physical problems due to the stroke event	2.30	1.18		
Shock	Likert	Degree of shock experienced during the stroke event	2.79	1.59		
Helplessness	Likert	Degree of helplessness or lack of control experienced during the stroke event	3.54	1.20		
Retelling distress	Binary	Expressing current distress when providing details of the stroke			2	2.0
Perceptual shift	Categorical	Participant reports that their emotions or perceptions changed from during the stroke event to when they reflect later				
Positive to negative					6	6.1
Negative to positive					2	2.0
Potential protective factors						
Positive expectancies	Binary	Mention having a positive outlook when asked about their stroke experience/recovery			12	12.2
Religious mentions	Binary	Citing religion or God in response to a question regarding the stroke experience			8	8.2
Concern for loved ones	Binary	Mentioning concern for others’ well‐being in relation to their own stroke event			7	7.1
Presence of others	Categorical	Participants description of who was present during their stroke event				
		Alone			31	31.6
		Other people, but mention feeling alone			6	6.1
		Strangers			4	4.1
		Coworkers/friends			11	11.2
		Family/caretaker/close loved one			46	46.9
Level of detail						
Impoverished response	Likert	Degree of auxiliary information provided in responses	2.80	0.91		
Somatic detail	Likert	Degree of details related to sensorial and physical experience	2.77	1.01		
Emotional detail	Likert	Degree of detail related to the emotional experience	2.28	1.13		

**TABLE 3:** Tests for associations between demographic characteristics (age and gender) and individual thematic codes

Demographic characteristics and code	Scale type	*r*	*t*(96)	χ^2^	*OR*	95% CI	*p*
Age							
Distress							
Fear	Likert	−.34					< .001
Negative consequences	Likert	−.24					.018
Perceived life threat	Likert	−.09					.364
Shock	Likert	−.10					.319
Helplessness	Likert	−.14					.175
Retelling distress	Binary				0.97	[0.89, 1.05]	.446
Potential protective factors							
Positive expectancies	Binary				1.05	[1.00, 1.10]	.039
Religious mentions	Binary				1.04	[0.98, 1.09]	.190
Concern for loved ones	Binary				0.98	[0.94, 1.02]	.348
Level of detail							
Impoverished response	Likert	.13					.197
Somatic details	Likert	−.03					.749
Emotional details	Likert	.03					.792
Gender							
Distress							
Fear	Likert		0.75				.457
Negative consequences	Likert		0.42				.676
Perceived life threat	Likert		0.20				.843
Shock	Likert		0.95				.346
Helplessness	Likert		0.40				.691
Retelling distress	Binary			(1, *N* = 98) = 2.51			.113
Perceptual shift	Categorical			(2, *N* = 98) = 0.09			.955
Potential protective factors							
Positive expectancies	Binary			(1, *N* = 98) = 0.74			.390
Religious mentions	Binary			(1, *N* = 98) = 1.09			.296
Concern for loved ones	Binary			(1, *N* = 98) = 0.01			.910
Presence of others	Categorical			(4, *N* = 98) = 4.72			.317
Level of detail							
Impoverished response	Likert		0.49				.625
Somatic details	Likert		0.13				.896
Emotional details	Likert		0.38				.706

*Note*: CI = confidence interval.

Two binary codes assessed aspects of the way in which participants reported distress, as this could have relevance for subsequent PTSD (Table 2). Although most participants referred to feelings of distress, the time course of these feelings varied for some. Indeed, nearly 10% of participants (*n* = 8) described a perceptual shift in their emotions from the time of the stroke/TIA to their current appraisal; of note, the mean time from study enrollment during hospitalization to the completion of the standardized trauma interview was 1.74 days (*SD* = 2.34, range: 0–13). Among participants who mentioned experiencing a shift in perception, the vast majority (*n* = 6) described low levels of distress related to their stroke/TIA initially, followed by elevated distress at a later point (e.g., during hospitalization). For example, when asked whether they thought they might be permanently disabled or die, one participant stated “No, but I did today.” Additionally, only two individuals explicitly mentioned current feelings of distress when retelling the events of their stroke/TIA experience as part of the standardized trauma interview. When asked how horrified or shocked they felt, one participant stated, “Yes. I still am. I can't let myself think or else I'll get too nervous now.”

#### Potential protective factors

Although the standardized trauma interview did not include questions related to potential protective factors or coping strategies, our review of the literature indicated these elements are relevant for PTSD, and we were interested in unprompted mentions of these factors (Figure 1). Indeed, more than one quarter of participants (*n* = 25) referenced a variety of topics that could be thought of as protective with respect to PTSD risk in the aftermath of stroke/TIA. The theme of potential protective factors encompassed codes related to positive expectancies, religious mentions, the presence of others, and concern for loved ones (see Table 2 for definitions and frequencies). The most frequently reported potential protective factor was positive expectancies, with 12 individuals noting a sense of optimism or self‐efficacy in response to the stroke/TIA experience. For example, when one participant was asked if they thought they would be permanently disabled or that they might die at the time of the stroke/TIA event, they responded, “No, no, no. I never think or thought negative like that.” Additionally, another individual stated, “No, not at all, I'm a strong man.” Numerous responses (*n* = 8) also included mentions of religion, including individuals noting a sense of assurance that they would survive the event, such as, “I didn't feel fear because I had faith in God” and “I knew God wasn't ready for me yet.”

All participants were asked about the presence of others during their stroke/TIA event, and most individuals reported they were with their family, caretaker, or a close loved one (e.g., a best friend; *n* = 46) or alone (*n* = 31). A minority of participants indicated being with coworkers or friends (*n* = 11) or strangers (*n* = 4). Additionally, six participants stated they felt alone despite others being present. For example, one participant noted, “No, I was alone…well, my husband was home, but I was alone in the room and going through it alone.” The review of responses also identified seven individuals who expressed concern for loved ones above concern for themselves when asked about their fear surrounding the event, including the statement, “My instincts were just to make sure that my kids were okay.”

#### Level of detail

This theme encompassed codes regarding the manner in which participants responded to the interview questions, including the extent to which their responses were detailed versus impoverished and whether they provided emotional and somatic details—elements relevant to PTSD risk (Figure 1; for full descriptions and descriptive statistics for codes, see Table 2). Participant responses ranged from single‐word answers for nearly all questions to paragraphs‐long descriptions, and this prompted the development of the impoverished response code, representing the degree of detail included in participant responses rated on a 5‐point Likert scale. The average impoverished response score was 2.89, with higher scores indicating more detail. This response scale was normally distributed (Supplementary Figure S1).

Participants also varied in the degree to which they included somatic and emotional details in their responses. For example, when describing the stroke/TIA event, some participants included detailed descriptions of the somatic experience of the stroke; one participant noted, “I woke up with a really bad headache. I had eye pain—my left eye—and thought it was a migraine.” Others were focused on the stress of the emotional experience of the stroke. For instance, one participant described their experience during the event in the following way: “I started feeling overwhelmed… feeling like I wasn't connecting to people.” Finally, some participants gave details about both the somatic and emotional experiences of the stroke/TIA event, such as, “I felt like I was sinking in bed. I didn't have feeling in one side…that was very concerning, that's when I was scared.” Scores for somatic detail were normally distributed, and the average somatic detail score was 2.77. The average level of emotional detail was 2.20, and scores had a positively skewed distribution (Supplementary Figure S1).

### Comparative analysis

Following the narrative analysis, we performed a comparative analysis to test for differences in codes across select demographic characteristics relevant to stroke‐induced PTSD risk, namely age and gender. Age had small‐to‐medium negative correlations with scores on two of the five distress‐related codes (Table 3; Supplementary Figure S2), including fear, *r* = −.34, *p* < .001, and negative consequences, *r* = −.24, *p* = .018. Of the binary codes related to potential protective factors, age was significantly associated with positive expectancies such that the odds of having positive expectancies increased by 5.2% for every additional year of age, odds ratio (*OR*) = 1.05, 95% confidence interval (CI) [1.00, 1.10], *p* = .039 (Table 3; Supplementary Figure S2). No other significant associations were observed for age, and there were no gender differences for any of the codes (Table 3; Supplementary Figure S3).

## DISCUSSION

In this study of trauma narratives in the acute aftermath of a stroke/TIA event, three superordinate themes were identified through an abductive thematic analysis: (a) distress (i.e., codes regarding fear, shock, perceived life threat, negative consequences of the stroke, helplessness, perceptual shift, and retelling distress), (b) potential protective factors (i.e., codes regarding positive expectancies, religious mentions, the presence of others, and concern for loved ones), and (c) level of detail (i.e., codes regarding impoverished response, emotional detail, and somatic detail). These themes were not only informed by the extant literature but also by a review of interview responses.

Distress was the most extensive theme identified in the review of the trauma narratives, and aspects related to distress were assessed using seven codes. Distress during and in the acute aftermath of a traumatic event, although no longer a diagnostic criterion for PTSD, is considered a significant risk factor for the development of PTSD (Vance et al., 2018). Although experiencing medical events such as stroke can be highly distressing for many individuals, our results suggest that individuals report a wide range of reactions to a stroke/TIA event. Consistent with the notion that stroke/TIA events can be sudden and unpredictable, most participants reported moderate or higher ratings for the helplessness code. In contrast, frequencies for scores on the fear and shock codes had U‐shaped distributions, with the most common scores falling at the maximum and minimum of the scales. Additionally, approximately one in six participants did not report fear, shock, or perceived life threat. Indeed, many participants in this study described being unaware that they were experiencing stroke/TIA prior to their diagnosis during hospitalization; this lack of awareness may have potentially contributed to minimal distress as the stroke/TIA event unfolded. This type of extended unawareness of an ongoing threat may be a more unique characteristic of medical traumatic events and merits consideration in future research on the psychological consequences of these experiences.

Our analysis of the trauma narratives also revealed a number of unprompted responses that could be considered potentially protective for the development of PTSD poststroke. Positive expectancies were the most common potential protective factor identified in this study and, similarly, are the most well‐represented in the extant literature on protective factors for the development of PTSD (Gallagher et al., 2020). Religious mentions were considered a potential protective factor, as the religious mentions identified in the present study often included a sense of reassurance that the individual would survive the event. Although previous research using linguistic analysis found that higher numbers of religion‐related words were associated with more chronic PTSD symptoms (D'Andrea et al., 2012), this finding may have been driven by expressions of distress that included a religion‐related word (e.g., “Oh, my God”). This suggests that considering the content of religious‐related statements and the context in which they are used may be important for research examining associations with PTSD‐related outcomes. The presence of others during the stroke/TIA event was included as a potentially protective factor as an indicator of social support. However, recent research has found that the presence of “close others,” compared to non–close others, in the emergency department during an acute coronary event is associated with increased feelings of distress and anxiety (Cornelius et al., 2020, 2019). Relatedly, concern for loved ones was also identified as a potential protective factor in the present study, as it could be a positive motivator for recovery. However, this theme has not been well‐examined in the PTSD literature, and more research is needed to assess whether this sentiment is related to subsequent distress and PTSD symptom development.

In addition, we examined both the extent to which responses were detailed versus impoverished and the degree of emotional and somatic content in the trauma narratives using the codes that comprised the level of detail theme. Numerous reviews of the literature regarding the association between trauma narratives and PTSD have concluded that more somatic‐ or emotion‐related word use within trauma narratives consistently predicts higher levels of PTSD symptoms (Crespo & Fernández‐Lansac, 2016; O'Kearney & Perrott, 2006). In the present study, the inclusion of somatic details in participants’ trauma narratives was prevalent despite many of the prompts being related to the emotional experience of the stroke/TIA. Existing theories on medically induced PTSD, such as the enduring somatic threat model, highlight the importance of somatic cues given the internal source of the threat associated with medical trauma (Edmondson, 2014). As a result, the level of somatic detail included in a trauma narrative may be especially important in the prediction of medically induced PTSD. We chose to include an impoverished response code to characterize the distinct response style of some individuals who provided single‐word answers for nearly all questions. Although associations between themes were not tested in this study, we noted that these single‐word answers often related to the denial of distress‐related experiences. However, this observation does not necessarily rule out distress and, alternatively, may be indicative of possible avoidance—a key symptom of PTSD. Indeed, one study found that trauma narratives with a lower total word count were associated with higher levels of avoidance‐related PTSD symptoms, whereas no associations between word count and hyperarousal, reexperiencing, or total PTSD symptoms were observed (Lindblom & Gray, 2010).

In addition to documenting and describing these themes in the narratives, we explored the extent to which age, a factor relevant to stroke‐induced PTSD, might be related to the codes that emerged from the narratives. Age was negatively associated with scores for codes related to fear and negative consequences, and older age predicted a higher likelihood of mentioning a positive outlook on one's stroke/TIA experience or recovery. These findings indicating that individuals with more advanced age cite less poststroke distress are consistent with other research that has demonstrated associations between younger age and a higher risk of developing PTSD symptoms following a sudden medical event (Edmondson et al., 2012; Garton et al., 2017; Goldfinger et al., 2014; Kronenberg et al., 2021). Despite established associations between advanced age and poorer functional recovery following a stroke (Knoflach et al., 2012), older age may also be related to several protective factors, including more financial stability and more preparedness for, and, thus, less perceived threat during, an acute cardiovascular event. This latter hypothesis is supported by findings from a study of patients evaluated for acute coronary syndrome in which the authors found that the association between younger age and posttraumatic stress symptoms was partially explained by elevated perceptions of threat (Meli et al., 2018). Further research is needed to examine how the coded responses to the trauma interview relate to other measures of acute posttraumatic stress and the risk for subsequent PTSD.

This study also examined potential gender differences in the codes identified in the narratives and found no significant differences. The association between female gender and PTSD risk has been well established in the general population; the lifetime prevalence of PTSD has been shown to be approximately twice as high in women (12.8%) as in men (5.7%; Kilpatrick et al., 2013). However, findings related to gender differences for stroke‐induced PTSD are less consistent, with some studies demonstrating an association between female gender and poststroke PTSD status or symptoms (Bruggimann et al., 2006; Kronenberg et al., 2021) and others not (Goldfinger et al., 2014; Merriman et al., 2007). Gender differences have also been observed in both the qualities of traumatic events to which men and women are exposed and the ways in which they are socialized to respond to such events, both of which may have implications for the PTSD risk (Street & Dardis, 2018; Tolin & Foa, 2006). However, there are several aspects of a stroke/TIA event that may result in less prominent gender differences in these PTSD‐relevant processes. For example, perceived physical strength may be a more salient protective factor against harm during a traumatic event for men compared to women, but physical strength is often greatly reduced during a stroke. Additionally, the buffer of more perceived control in response to a traumatic event that has been observed more frequently in men relative to women in previous research (Olff et al., 2007) may be nullified by the internal nature of the threat associated with a stroke/TIA, resulting in similar experiences of helplessness, perceived life threat, and negative consequences during a sudden life‐threatening medical event (e.g., stroke/TIA). Such explanations could underlie the similar frequency distributions of distress‐related code scores observed for men and women in the current study.

There were several limitations to the present study. Our quantitative findings were correlational and exploratory, and they were based on a small convenience sample. Further research in larger samples is needed to replicate these results. Additionally, the trauma narratives examined in the study were provided in response to structured interview questions, potentially limiting the variability in responses relative to other methods of eliciting trauma narratives, such as narrative reliving (Payne, 1987). The trauma narratives were also documented through bedside notes rather than recorded and transcribed verbatim, which may have limited the precision of the narrative review given potential discrepancies between the written notes and the exact wording participants used. However, this study also has notable strengths, including our relatively large and diverse sample, which included individuals with a wide range of sociodemographic characteristics. Additionally, trauma narratives were provided during hospitalization and within days of the stroke/TIA event, likely increasing an individual's ability to accurately recall and recount their experience. Finally, the abductive thematic approach provided structure to our methodological approach and enhanced our identification of themes rooted in both empirical and theoretical evidence.

This study is the first of its kind to perform a narrative analysis of PTSD‐relevant experiences recalled by individuals in the acute period following a suspected stroke/TIA. The analysis revealed a wide range of variability in levels of distress and detail levels in responses, notable protective themes that were expressed by some individuals during their stroke/TIA, some differences across age, and overall consistencies across gender. This research expands understanding of the ways in which individuals react to medical trauma such as a stroke/TIA and sets the stage for the identification of acute responses to a traumatic event that may have implications for developing medically induced PTSD. Future research is needed to determine whether these acute responses can be used to identify patients at risk for PTSD development and whether they may serve as potential PTSD prevention and intervention targets.

## OPEN PRACTICES STATEMENT

The study reported in this article was not formally preregistered. The data have not been made available on a permanent third‐party archive; requests for the data can be sent via email to the lead author. The complete interview and codebook used in this study are included in the Supplemental Online Material associated with this article. Requests for data access can be sent to the corresponding author at cmeinhausen@ucla.edu.

## AUTHOR NOTE

This work was supported by the National Heart, Lung, and Blood Institute (R01HL132347, K01HL130650, R01HL139614) and the National Institute of Mental Health (T32MH015750), both of the National Institutes of Health.

## Supporting information

Supporting Information

Supporting Information

Supporting Information

Supporting Information
