# Supplementary material for: Development of a codebook for the narrative analysis of in‐hospital trauma interviews of patients following stroke
Source: J Trauma Stress. 2024 Nov 1;38(1):86–98. doi: 10.1002/jts.23106 (PMC11791883; doi:10.1002/jts.23106)
Supplement: Supplementary file 1 — Supporting Information [file JTS-38-86-s004.docx]

**Stoke/TIA Trauma Interview Qualitative Codebook**

For use with the 15-item trauma interview in which REACH Stroke study participants answered questions and described the experience of their stroke/TIA event during skin conductance measurement. All responses should be considered when making the rating for each code. Please refer to example responses for each code when scoring responses.

**1.**

| Code: | **Fear Score (Ordinal Scale)** |
| --- | --- |
| Brief definition: | Level of fear experienced during the stroke |
| Full definition: | Spectrum of how much or little fear (including feeling terrified or afraid) the participant expressed related to the stroke event. On one end of the spectrum, they do not endorse feelings of fear. On the other end of the spectrum, they describe feeling very high levels of fear during the stroke event. Someone who says “No” vs. “I did not feel afraid or terrified” would both receive a score 1. |
| Example Responses: | **Q12. During the stroke/TIA event, how terrified or afraid did you feel?**  1- “Not at all”  2- “Not terrified but curious. I didn't know what was going on”,  3- “I was concerned about getting through it, but I wasn't afraid.”, “a little afraid”  4- “I was scared. Oh yea, like I said I was stressed”  5- “I was very terrified, I couldn't stop thinking “stroke, stroke, stroke"” |

**2.**

| Code: | **Helplessness (Ordinal Scale)** |
| --- | --- |
| Brief definition: | Degree of helplessness felt during the stroke event |
| Full definition: | The degree to which the patient felt a sense of helplessness or control during the stroke event on a scale of 1-5, high end of the helplessness spectrum may be descriptions of feeling completely out of control. Pay attention to mentions of proactive behavioral measures taken by the participant. |
| Examples: | **Q10. During the stroke/TIA event, how helpless did you feel?**  1- “Not at all”  2- “I was more worried and wondered what had happened”  3- “I didn't feel totally helpless because I was aware the whole time”, “Not completely, 5/10”  4- “Pretty helpless. I'm a pretty independent person so I feel very helpless.”  5- “Very! First time in my life that I had to ask for help. I couldn't put on my socks, I needed help to get dressed, I've been needing help to shower, I've been needing a lot for help. First time I've needed this much help.”, “Very, very helpless” |

**3.**

| Code: | **Perceived Life Threat (Ordinal Scale)** |
| --- | --- |
| Brief definition: | Degree of perceived life threat during the stroke event |
| Full definition: | The degree to which patients perceived the stroke event as potentially life threatening on a scale of 1-5. |
| Example Response: | **Q9. At the time of the stroke/TIA event, did you think you would be permanently disabled or that you might die?**  1- “No I didn't think of any of that”  2- “I did think about it, but didn't dwell on it”, “I don’t know”, “I hope not”  3- “I was optimistic, but of course concerned.”  4- “I was worried I was going to die”, “I thought this might be the big one.”  5- “Thought I'd die, thought I was gone” |

**4.**

| Code: | **Negative Consequences of the Stroke (Ordinal Scale)** |
| --- | --- |
| Brief definition: | Mentioning long-term negative consequences while recalling details of the stroke. |
| Full definition: | Spectrum of how much or how little the participant anticipates long-term physical problems due to the stroke event. On one end of the spectrum, they don’t believe so. On the other end of the spectrum, they do believe they will have long-term problems or already cite problems. On the middle of the spectrum may include responses such as them hoping to not have long-term problems or that they don’t know. |
| Example Response: | **Q9. At the time of the stroke/TIA event, did you think you would be permanently disabled or that you might die?**  1- “No, I didn't give it any thought”  2- “I did think about it, but didn't dwell on it”  3- “I thought it was something serious, that maybe I’d stay like that with my face drooped. I was paranoid”, “I hope not”  4- “As much as I believe in God, and though I’m currently very much so not one to get upset, but I was very worried about being disabled and dying.”  5- “Yea forever paralyzed. I couldn't imagine it. I just kept thinking who's going to take care of me if I was paralyzed” |

**5.**

| Code: | **Shock Score (Ordinal Scale)** |
| --- | --- |
| Brief definition: | Level of shock described by the patient in relation to the stroke. |
| Full definition: | Expressing shock in relation to the stroke. On one end of the spectrum, they were not shocked at all that they were having a  stroke. On the other end of the spectrum, they were extremely shocked that they were having a stroke. |
| Example Response: | **Q11. During the stroke/TIA event, how horrified or shocked did you feel?**  1- “I wasn’t shocked because I had a feeling. I knew something was  wrong.”, “No I didn't feel like that”  2- “Not strongly but yes a little”  3- “I was surprised, but I wasn't shocked.”  4- “I was shocked but, it was controlled”  5- “Yea very shocked.” |

**6.**

| Code: | **Emotional Detail Score (Ordinal Scale)** |
| --- | --- |
| Brief definition: | Emotional details related the stroke event |
| Full definition: | Spectrum of how much emotional detail they provide when describing the stroke event. On one end of the spectrum, the experience of the stroke is described generally and matter-of-factly with very little detail about their emotional experience. At the other end of the spectrum, they provide extensive information regarding their emotional experience when describing the event. |
| Example Response: | **Q1. Please briefly describe the event that happened to you when you first felt that something was wrong during the stroke/TIA event.**  1- “First I started to feel lightheaded, kind of faint, and then I felt unbalanced.”  2- “I realized I couldn't focus on an object right in front of me, like I was losing my sight, and I couldn't even see my left hand - that's when I knew something was wrong.”  3- “It started when I was confused and felt unstable walking and it was difficult talking. I didn't know what was happening but I thought I was just feeling tired. It was strange. I felt weird, strange. I was at a friend's dinner party. They were throwing a dinner party for me and my friend noticed I was walking strange and said I should go rest, so I was going to go to the hotel to rest, try to get some sleep because I thought maybe I was just tired. I had just gotten off a flight earlier that day so I thought I was just tired.”  4- “My left hand started going numb on my way to work and I quickly thought that it was an embolism but I didn't want to think that so I ignored it, just thought I slept wrong and went on with work. The next day, it was the same thing, for about 2-3 hours, and day after day it kept happening. I was getting scared of course and then on Tuesday my face went side ways (droop) and I was really frightened/surprised/scared. Then my wife noticed it and we came to the hospital’  5- “I was on the computer and then I didn't feel alright. It was a funny feeling in my head and my first thought was that I hope it's not an aneurysm. I then got up to go to the bathroom around noon and then my partner returned from doing laundry and asked if I was alright. That's when I started talking gibberish and she thought I was joking at first but I really couldn't communicate. I wasn't afraid but I thought oh this is it. Honestly I don't want to live if I can't talk. I was trying not to go to the doctor but before I knew it my partner was on the phone with 911.” |

**7.**

| Code: | **Somatic Detail (Ordinal Scale)** |
| --- | --- |
| Brief definition: | Degree of somatic descriptions in participant’s account of the stroke event |
| Full definition: | Sensorial/perceptual details related to the somatic experience of the event stroke rated on a scale of 1-5. Somatic descriptions include any description of the physical sensations perceived during the event. This is specifically for somatic descriptions in relation to the index stroke event. Focus on how much attention is paid to physiological symptoms and sensations, rather than injuries related to the stroke. To what extent are they using somatic descriptors in their narrative describing the stroke event? |
| Examples: | **Q1. Please briefly describe the event that happened to you when you first felt that something was wrong during the stroke/TIA event.**  1- “I started dropping things and then fell in the shower”  2- “I woke up and had to use the bathroom, but then I realized that I couldn't walk. My bathroom is next to my bedroom, so I got there by holding onto things and moving slowly.”, “I woke up and things were skewed. I couldn't focus on anything. I went up to go do something and couldn't focus on anything. I went to do laundry, went to the machine, put the stuff in and it didn't get done. I didn't do it. I don't know I couldn't do things. I never got to it. I called my best friend and I'm not sure I went down I guess and my super brought me up to my apartment. I think my friend went up then and said to stay in bed. It was really strange, like nothing was going to work right. I just knew I had to be still and come here. Something in me said stay here, my friend tried to get dressed to bring me to the ER, and then I seemed OK but it was disturbing. I don't know what was going on.”  3- “I was at home walking to the bathroom and stumbled and felt like I was outside of my body”  4- “I woke up really nauseous and vomited, and I felt like my head was going to explode. So I took my migraine medication and was stressed about having to call out of work, so I sent a few emails.”  5- “I was laying in bed with my girlfriend watching TV when I got a shooting pain in the front of my head like by my forehead. It was like a sledgehammer feeling. I rolled on my back and I was going to lay there for a minute but my girlfriend said I had a small seizure.” |

**8.**

| Code: | **Impoverished Response Score (Ordinal Scale)** |
| --- | --- |
| Brief definition: | The degree of additional information the response provides |
| Full definition: | Spectrum of how much or little information and detail is included in the responses. On one end of the spectrum, they respond with single word answers to most questions. On the other end of the spectrum are responses that include significant detail and describe things like their thought process and additional context. This is specifically in comparison to the typical length of answers for that question. |
| Example Response: | **Q10. During the stroke/TIA event, how helpless did you feel?**  1- Single word answers for nearly all questions  2- Some single word answers, when they elaborate, they don’t include much more detail “I have trust in my brother”  3- “I've never felt completely helpless, I'm in control of things. I just had to figure it out.”, “a lot - I had no strength at all, of course I felt helpless”  4- “I did and I didn't. I didn't know what was happening, but I fought it, I was trying to be calm and I was able to be calm and fight it”  5- “Very! First time in my life that I had to ask for help. I couldn't put on my socks, I needed help to get dressed, I've been needing help to shower, I've been needing a lot for help. First time I've needed this much help.” |

**9.**

| Code: | **Perceptual Shift (Categorical)** |
| --- | --- |
| Brief definition: | When feelings change over time. |
| Full definition: | Describes a situation in which the participants report that their emotions or perceptions changed from during the stroke event to when they reflect later. Code 0 - no change, 1 - positive to negative, 2 - negative to positive |
| Examples: | 0- No mention of changing feelings  1- “No but I did today”, “No, because I didn't know what happened, When I learned I what happened I was worried.”  2- “Pretty helpless but once it passed I just thought that was weird and didn't give it much attention.”, “Very scared but again only during it. Once it passed, like 4 minutes later, I just thought, that was weird.” |

**10.**

| Code: | **Presence of Others (Categorical)** |
| --- | --- |
| Brief definition: | Description given by participants of who was present during their stroke event |
| Full definition: | Detail regarding who was present during the stroke (before hospitalization) described by the patient. Could be family, coworkers, friends, or strangers. Responses that mention that they felt alone despite other people being present should receive a 2 (e.g., “No I was alone. Well, my husband was home, but I was alone in the room and going through it alone”) while unknown others (e.g., people at a bus stop) would receive a 3. |
| Example Response: | **Q6. Was anyone else with you when you had the stroke/TIA event?**  1- Alone  2- Other people but mention feeling alone  3- Strangers  4- Coworkers/Friends  5- Family/Caretaker/Close loved one (e.g., best friend, partner) |

**11.**

| Code: | **Religious Mentions (Binary)** |
| --- | --- |
| Brief definition: | Citing religion or God in relation to the stroke experience. |
| Full definition: | Describes when a participant cites religion or God in response to a question posed regarding the stroke experience. (0=no, 1=yes) |
| Examples: | “God told me I had to do something. So I crawled along the bed to the phone and I called 911 and they took me to the hospital.” (Q1)  “Only God knows” (Q8)  “Not worried, already on disability, not going to die. I knew God wasn't ready for me yet.” (Q9)  “No, it's what God wants” (Q12) |

**12.**

| Code: | **Concern for Loved Ones (Binary)** |
| --- | --- |
| Brief definition: | Citing concern experienced for loved ones. |
| Full definition: | Describes when a participant mentions feeling concern for others, particularly loved ones, in relation to their own stroke event. (0=no, 1=yes) |
| Examples: | “I thought for a minute that I was going to die in front of my children. How they would react. How scared they would be. That worried me” (Q9)  “Not helpless, I was just worried about my baby” (Q10)  “Not really, my instincts were just to make sure that my kids were okay” (Q11)  “No, but I was scared for my family after it happened.” (Q12) |

**13.**

| Code: | **Positive Expectancies (Binary)** |
| --- | --- |
| Brief definition: | Expressing an optimistic outlook on life when describing the stroke event. |
| Full definition: | When asked about their stroke experience, the individual mentions having a generally positive outlook. Examples of optimism include mentioning having a positive outlook, seeing the glass as half full, or a sense of personal fortitude in relation to the stroke experience or their anticipated recovery. (0=no, 1=yes) |
| Examples: | “No, I always see the glass as half full” (Q9)  “No, no, no I never think or thought negative like that” (Q9) |

**14.**

| Code: | **Retelling Distress (Binary)** |
| --- | --- |
| Brief definition: | Feeling distressed when discussing details of the stroke. |
| Full definition: | Expressing feelings of distress when they are asked to remember details of the stroke. This is specifically mentioning distress related to the experience of retelling the stroke details, not distress they experienced during the stroke. (0=no, 1=yes) |
| Examples: | “I can’t let myself think or else I’ll get too nervous now.” (Q11) |
