# Supplementary material for: Development of a codebook for the narrative analysis of in‐hospital trauma interviews of patients following stroke
Source: J Trauma Stress. 2024 Nov 1;38(1):86–98. doi: 10.1002/jts.23106 (PMC11791883; doi:10.1002/jts.23106)
Supplement: Supplementary file 2 — Supporting Information [file JTS-38-86-s001.docx]

**SUPPLEMENTARY FIGURE S1**

*****Histograms of score distributions for the Likert scale codes (n=8) related to A) Distress and B) Level of detail*

**A)**

**B)**
