# Supplementary material for: Development of a codebook for the narrative analysis of in‐hospital trauma interviews of patients following stroke
Source: J Trauma Stress. 2024 Nov 1;38(1):86–98. doi: 10.1002/jts.23106 (PMC11791883; doi:10.1002/jts.23106)
Supplement: Supplementary file 3 — Supporting Information [file JTS-38-86-s002.docx]

**SUPPLEMENTARY FIGURE S2**

*Graphs of significant associations between age and codes including: A) Scatterplot of age and fear code scores (r = -.34; p < .001), B) Scatterplot of age and negative consequences code scores (r = -.24; p = .018), C) Scatterplot comparing ages of individuals who did and did not mention positive expectancies*

**A) B)**

**C)**
