# Supplementary material for: Development of a codebook for the narrative analysis of in‐hospital trauma interviews of patients following stroke
Source: J Trauma Stress. 2024 Nov 1;38(1):86–98. doi: 10.1002/jts.23106 (PMC11791883; doi:10.1002/jts.23106)
Supplement: Supplementary file 4 — Supporting Information [file JTS-38-86-s003.docx]

**SUPPLEMENTARY FIGURE S3**

*Histograms comparing the frequency of Likert scale scores of men (n = 44) and women (n = 54) for: A) Distress-related codes, B) Level of detail-related codes*

**A)**


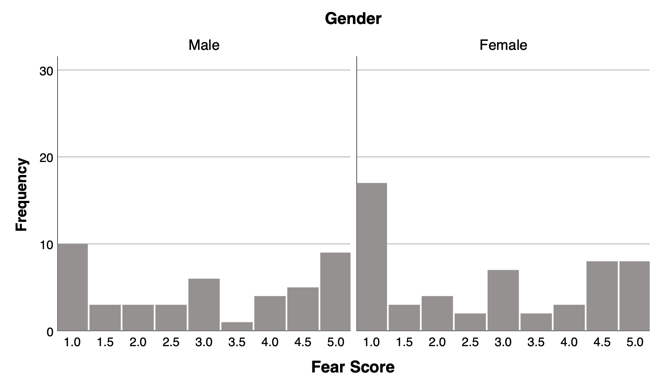

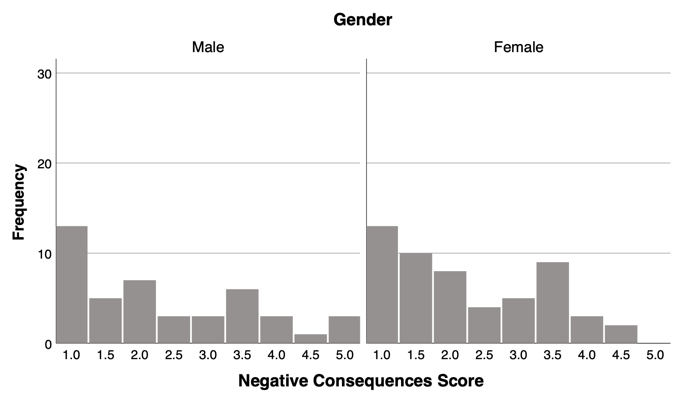

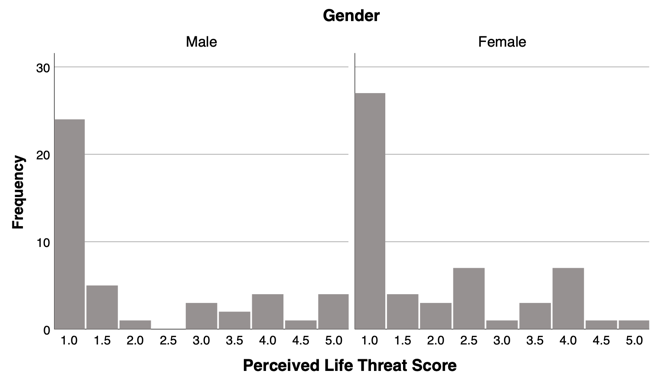

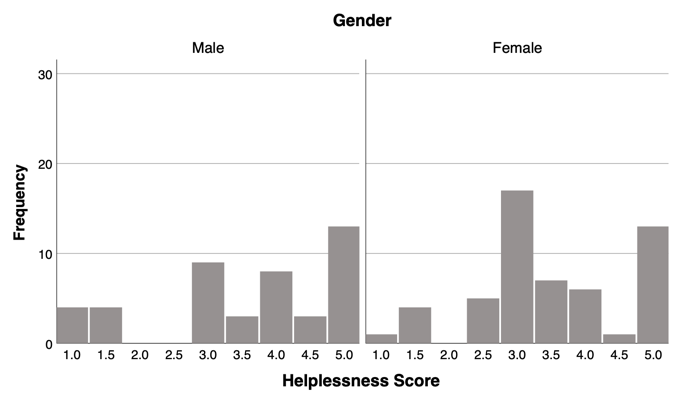


M= 2.35 M= 2.25

SD= 1.29 SD= 1.09

M= 3.02 M= 2.79

SD= 1.54 SD= 1.57

M= 3.59 M= 3.49

SD= 1.34 SD= 1.09

M= 2.04 M= 1.99

SD= 1.45 SD= 1.23

**
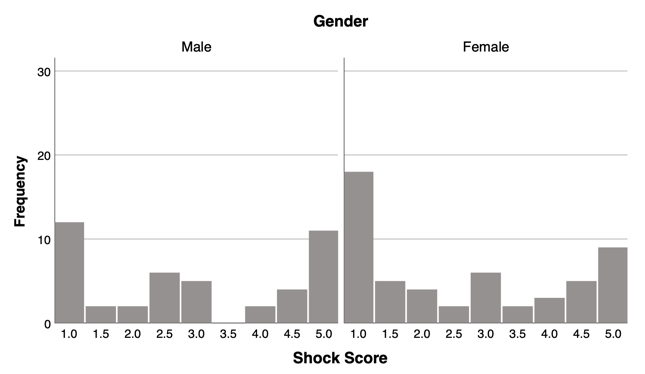
**

M= 2.95 M= 2.65

SD= 1.61 SD= 1.58

**B)**


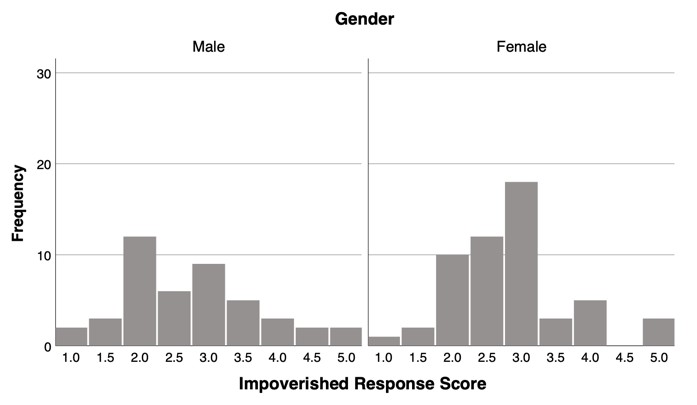

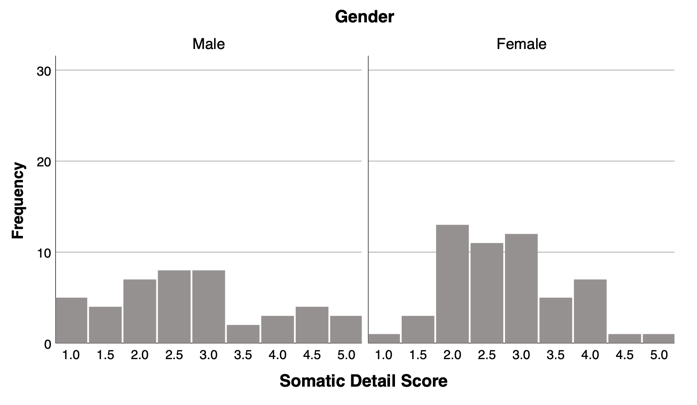

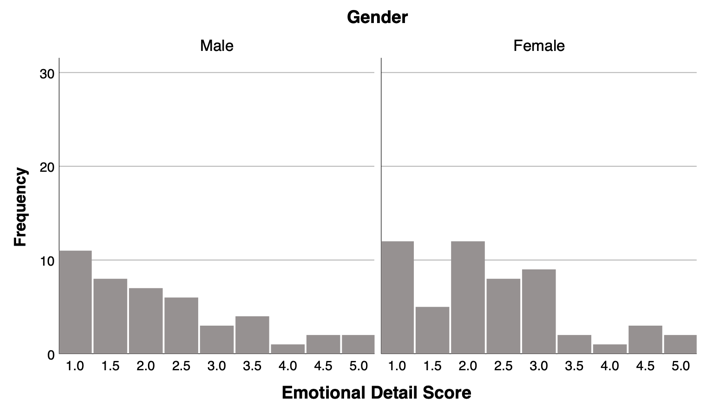


M= 2.75 M= 2.84

SD= 0.99 SD= 0.85

M= 2.22 M= 2.32

SD= 1.17 SD= 1.10

M= 2.75 M= 2.79

SD= 1.18 SD= 0.85
